# Regionally Distinct Modes of Intestinal–Systemic Vascular Connections in Developing Zebrafish

**DOI:** 10.3390/jdb14030037

**Published:** 2026-08-14

**Authors:** Akihiro Urasaki, Koichi Kawakami, Osamu Nakagawa

**Affiliations:** 1 Department of Molecular Physiology, National Cerebral and Cardiovascular Center Research Institute, 6-1 Kishibe-Shimmachi, Suita 564-8565, Osaka, Japan; 2 Graduate School of Science, Technology and Innovation, Kobe University, 1-1 Rokkodai-cho, Nada-ku, Kobe 657-8501, Hyogo, Japan; 3 Engineering Biology Research Center, Kobe University, 1-1 Rokkodai-cho, Nada-ku, Kobe 657-8501, Hyogo, Japan; 4 Laboratory of Molecular and Developmental Biology, National Institute of Genetics, Mishima 411-8540, Shizuoka, Japan; 5 Department of Genetics, The Graduate University for Advanced Studies, SOKENDAI, Mishima 411-8540, Shizuoka, Japan; 6 Department of Genomic Medicine, National Cerebral and Cardiovascular Center Research Institute, 6-1 Kishibe-Shimmachi, Suita 564-8565, Osaka, Japan

**Keywords:** intestinal vascular development, anastomosis, supra-intestinal artery (SIA), dorsal aorta (DA), ALK1 signaling, activin A receptor like type 1 (*acvrl1*)

## Abstract

Connecting the intestinal vasculature with the pre-existing systemic circulatory system is vital for nutrient absorption and blood transport for detoxification in the liver. However, the cellular processes underlying this connection remain unclear. Using genetically modified zebrafish models and in vivo imaging, we visualized that the supra-intestinal artery (SIA) connected to the pre-existing dorsal aorta (DA) in two distinct regions, the anterior and posterior. We found that endothelial cells migrated out of the DA and formed the anterior SIA-DA junction, whereas endothelial cells sprouted from SIA and connected to the DA in the posterior region. Pharmacological and genetic analyses revealed that activin receptor-like kinase 1 (ALK1) signaling was required for the SIA-DA connections. The present study provides new insights into the cellular phenomena and regulatory mechanisms of intestinal vascular development.

## 1. Introduction

In early vertebrate vascular development, circulation is initially established in the trunk, followed by cerebral circulation for neural function, branchial or pulmonary circulation for respiration, and portal circulation for nutrient absorption [1]. The portal circulatory system is atypical in that it involves the entry of venous blood from the gastrointestinal tract into the liver, which differs from those of other organs such as the brain, kidney, and muscle [2]. Intestinal vasculature serves as the entry point to this unique system, where blood cells from intestinal vessels run through the portal, hepatic, and cardinal veins [3]. Toxic substances are initially sent to the liver for the conversion into non-toxic forms and then travel throughout the body [2]. Therefore, intestinal vessels play an essential role in nutrient absorption in the intestine and detoxification of toxic substances in the liver. However, the process by which the newly formed intestinal vasculature connects to the pre-existing systemic circulatory system during embryonic development remains poorly understood.

Recent advances in live imaging have further established zebrafish as a powerful model for investigating endothelial cell behaviors during vascular morphogenesis [4,5]. Subintestinal vein (SIV) formation has been extensively analyzed in zebrafish, providing a valuable model for studying intestinal vasculature. Photoconversion and live imaging experiments revealed that endothelial cells in the SIV are derived from the ventral posterior cardinal vein (PCV) [6]. Migration of endothelial cells from the PCV is imperative for SIV formation, which is regulated by vascular endothelial growth factor (VEGF) and bone morphogenetic protein (BMP) signaling [6,7,8]. Similarly, supra-intestinal artery (SIA), which supplies blood to the intestine, also originates from the ventral PCV. This structure emerges after the second day post-fertilization, which is also controlled by VEGF and BMP signaling pathways [6,7,9]. Such PCV-derived endothelial cells that contribute to the SIV and SIA formation in the intestinal region are defined here as intestinal endothelial cells. Despite these findings, the molecular mechanisms that govern the connection of intestinal vasculature to pre-existing vessels remain unclear.

Previous studies on activin receptor-like kinase 1 (ALK1), also known as activin A receptor-like type 1 (ACVRL1), revealed its indispensable role in vascular development. *Acvrl1*-deficient mice exhibit defective yolk sac angiogenesis and embryonic lethality due to impaired vascular maturation [10,11]. In zebrafish, *acvrl1* is expressed in cranial and major trunk vessels including the dorsal aorta (DA) [12,13]. *Acvrl1* mutants display cranial vascular malformations characterized by direct connections between arteries and veins without intervening capillaries [12,13] and exhibit embryonic lethality. However, the role of ALK1 signaling in the formation and connection of intestinal vasculature has not been investigated.

In this study, we examined the cellular processes underlying the connection between the intestinal vasculature and the pre-existing DA. We found that endothelial cells from the DA contribute to the anterior connection with the SIA, while intestinal endothelial cells form sprouts to connect with the DA in the posterior region. Using pharmacological and genetic approaches, we further demonstrate that ALK1 signaling is indispensable for the formation of SIA-DA connections during development. The present study highlights regionally distinct modes of intestinal–systemic vascular connections and provides insights into the regulatory mechanisms of vascular development.

## 2. Materials and Methods

### 2.1. Zebrafish Husbandry and Transgenic Lines

Zebrafish were bred and maintained under standard laboratory conditions. Embryos were obtained by natural mating and by hours post-fertilization (hpf) at 28.5 °C. Transgenic lines used were *Tg(fli1a:EGFP)^y1^* [14], *Tg(kdrl:GFP)^s843^* [15], *Tg(kdrl:HRAS-mCherry)^s916^* [11], *Tg(gata1:dsRed)^sd2^* [16], *Tg(fli1:H2B-mC)^ncv31^* [17], *Tg(UAS:lifeact-mCherry)* [18], *Tg(UAS:GFP)* [19] and *Tg(UAS:mRFP)* [19].

### 2.2. Generation of Genetically Modified Zebrafish Models

The gSAIzGFFD332A [*Tg(afap1l1b:Gal4)*] and gSAIzGFFD478A [*Tg(egfl7:Gal4)*] lines were generated by the *Tol2*-mediated Gal4 gene trap method [19,20]. *Tg(dll4:eGFP)* transgenic lines were created by using *Tol2* transposon system [21]. A zebrafish *acvrl1* mutant line was generated using the CRISPR/Cas9 system [22]. Vectors for customized guide RNAs (gRNAs) were constructed as described previously [22]. Plasmid pT7-acvrl1 was constructed by cloning the two annealed oligonucleotides acvrl1-f [5′–TAGGAGGTACGTAGCACTGTTCGG–3′] and acvrl1-r [5′–AAACCCGAACAGTGCTACGTACCT–3′]. The gRNAs (50 pg/embryo) and Cas9 mRNA (300 pg/embryo) were synthesized and injected into fertilized eggs as described previously [23]. The injected embryos were raised and crossed with wild-type fish. To detect the *acvrl1* mutant allele, genotyping PCR was performed with acvrl1-5′ [5′–CTGTGAAAAATTCCTTGCTCTGT–3′] and acvrl1-3′ [5′–CCCAGAAACGAGTAAGTGAGCTA–3′] primers. The PCR products were sequenced using an ABI PRISM3130 (Applied Biosystems, Waltham, MA, USA). The *acvrl1* mutant allele contains a 5-bp deletion in exon 4, resulting in a frameshift that introduces a premature termination codon.

### 2.3. Genotyping

PCR-based genotyping was performed to identify *acvrl1* mutants. Primers acvrl1-wt [TAGGAGGTACGTAGCACTGTTCGG] and acvrl1-5′ were used to detect the wild-type *acvrl1* allele, and primers acvrl1-mut [GAATAGCAGGTACGTAGCACTGC] and acvrl1-5′ were used for the mutant allele.

### 2.4. Inhibitor Experiments

Zebrafish embryos were treated from 60 to 84 hpf with the following inhibitors: 10 µM K02288 (Selleck Chemicals, Houston, TX, USA, S7359), 10 µM dorsomorphin homolog 1 (DMH1) (Sigma-Aldrich, St. Louis, MO, USA, D8946), 2.5 µM SU5416 (Sigma-Aldrich, S8442), 25 µM *N*-[*N*-(3,5-difluorophenacetyl)-*L*-alanyl]-S-phenylglycine tert-butyl ester (DAPT) (Sigma-Aldrich, D5942) and 20 mM 2,3-butanedione monoxime (BDM; Sigma-Aldrich, B0753). Embryos were incubated in the inhibitor solutions or dimethyl sulfoxide (DMSO) (control) at 27.5 °C, and the posterior SIA-DA connection was examined at 84 hpf.

### 2.5. Confocal and Time-Lapse Microscopy

Zebrafish embryos were anesthetized with 0.016% tricaine (ethyl 3-aminobenzoate, Sigma-Aldrich) and embedded laterally in 1.0% low-melting-point agarose (Invitrogen, Carlsbad, CA, USA) on a glass-bottomed dish. Imaging was performed using an Olympus FV3000 or FV1200 confocal microscope (Evident Corporation, Tokyo, Japan) with a 10× or 20× objective, and Z-stack images were acquired with a step size of 1–3 µm, followed by image analysis using Fiji software (ImageJ2 version 2.3.0/1.53t). Confocal images were acquired at the developmental stages indicated in the corresponding figure legends. Time-lapse imaging was performed from 38 to 58 hpf to visualize the anterior SIA–DA connection and from 71 to 83 hpf to visualize the posterior SIA–DA connection.

## 3. Results

### 3.1. Visualization of the Intestinal Circulatory System

**Figure 1:**
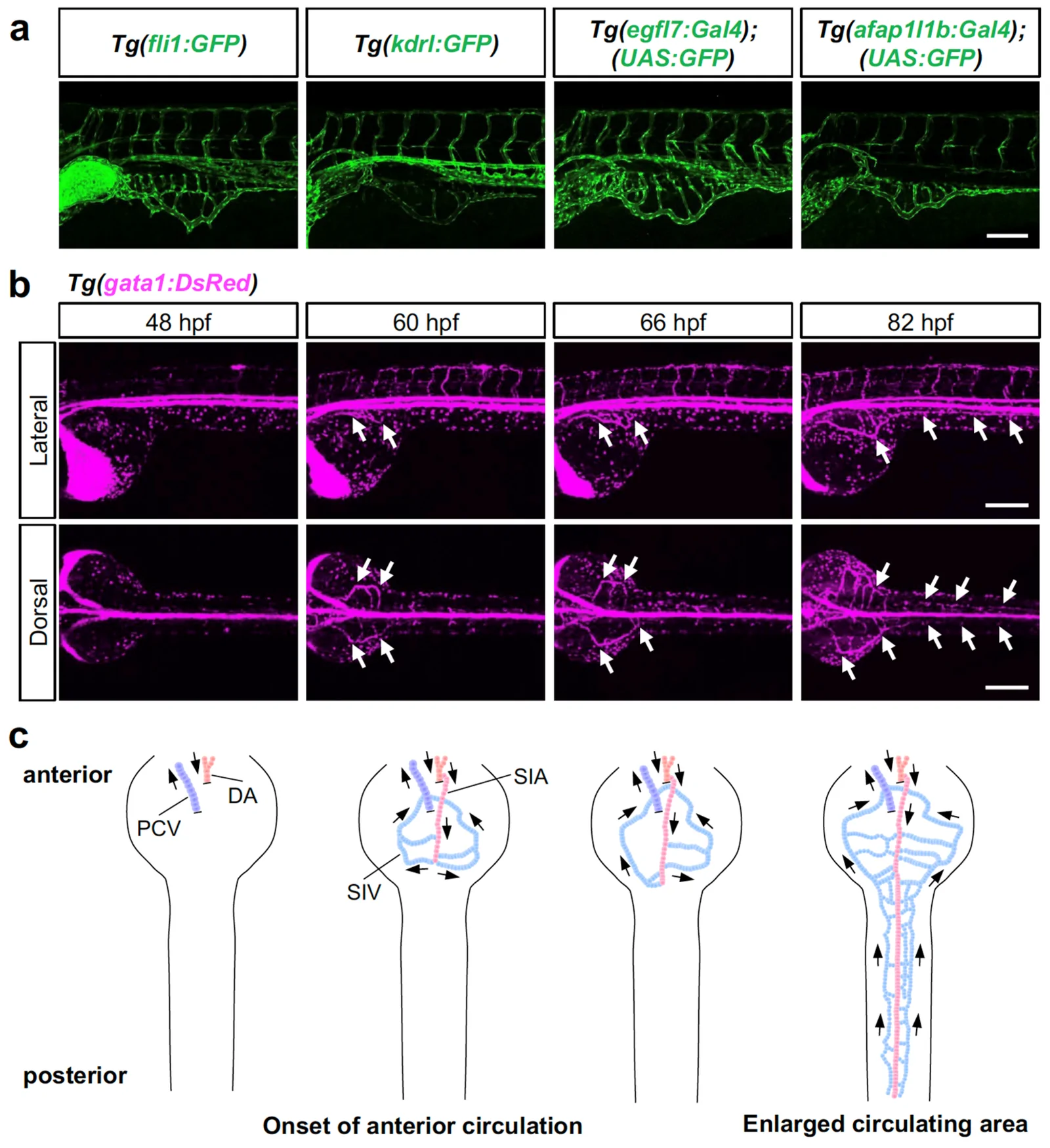
Visualization of the intestinal circulatory system. (**a**) Lateral-view confocal images of the intestinal and hepatic vasculature of 84 hpf *Tg(fli1:GFP)*, *Tg(kdrl:GFP)*, *Tg(egfl7:Gal4);(UAS:GFP)* and *Tg(afap1l1b:Gal4);(UAS:GFP)* embryos. The *Tg(fli1:GFP)* line has strong GFP fluorescence in surrounding tissues of intestinal vessels such as the pectoral fins, and the *Tg(kdrl:GFP)* line has very low fluorescence in the intestinal vessels. *Tg(egfl7:Gal4);(UAS:GFP)* fish expressed GFP in blood vessels including DA, PCV, ISV and intestinal blood vessels, whereas *Tg(afap1l1b:Gal4);(UAS:GFP)* fish highly expressed GFP in intestinal blood vessels. Scale bar: 50 μm. (**b**) Confocal images of the intestinal region of *Tg(gata1:DsRed)*. Upper panels show lateral views, and lower panels show dorsal views. The *Tg(gata1:DsRed)* line marked blood cells. DsRed signals were initially observed in the anterior region and then expanded to the posterior regions. Arrows indicate blood vessels with blood flow in the intestinal region. Scale bar: 50 μm. (**c**) Schematic diagram of developing intestinal circulation. Intestinal circulation started at approximately 60 hpf and expanded to the posterior region by 82 hpf.

The *Tg*(*fli1:GFP*) and *Tg*(*kdrl:GFP*) lines have been widely used to visualize vasculature in zebrafish. However, the *Tg*(*fli1:GFP*) line exhibited fluorescence interference in the pectoral fin, and the *Tg*(*kdrl:GFP*) line demonstrated low signal intensity in the intestinal vasculature at 84 h post-fertilization (hpf) (Figure 1a), which constrained the observation of posterior SIA-DA connections that formed at analogous developmental stages. We therefore introduced novel transgenic lines, *Tg(afap1l1b:Gal4)* and *Tg(egfl7:Gal4)*, which we found to better visualize intestinal blood vessels in combination with *Tg(UAS:GFP)* (Figure 1a). The *Tol2*-based gene trap construct was inserted into an intron of *egfl7* on chromosome 21 in the *Tg(egfl7:Gal4)* line and into an intron of *afap1l1b* on chromosome 21 in the *Tg(afap1l1b:Gal4)* line (Supplementary Figure S1). Heterozygous and homozygous fish of both transgenic lines were viable and fertile with no detectable phenotypic abnormalities.

To understand how circulation is established in intestinal vasculature, blood cells were visualized using *Tg(gata1:DsRed)* fish. Erythrocytes (gata1^+^ cells) were detectable in the intestinal vessels after approximately 60 hpf, and the circulation area extended to the posterior region by 82 hpf (Figure 1b,c). These observations indicated that intestinal circulation initially started anteriorly and gradually expanded posteriorly during development.

### 3.2. Connections Between SIA and DA in Two Regions

**Figure 2:**
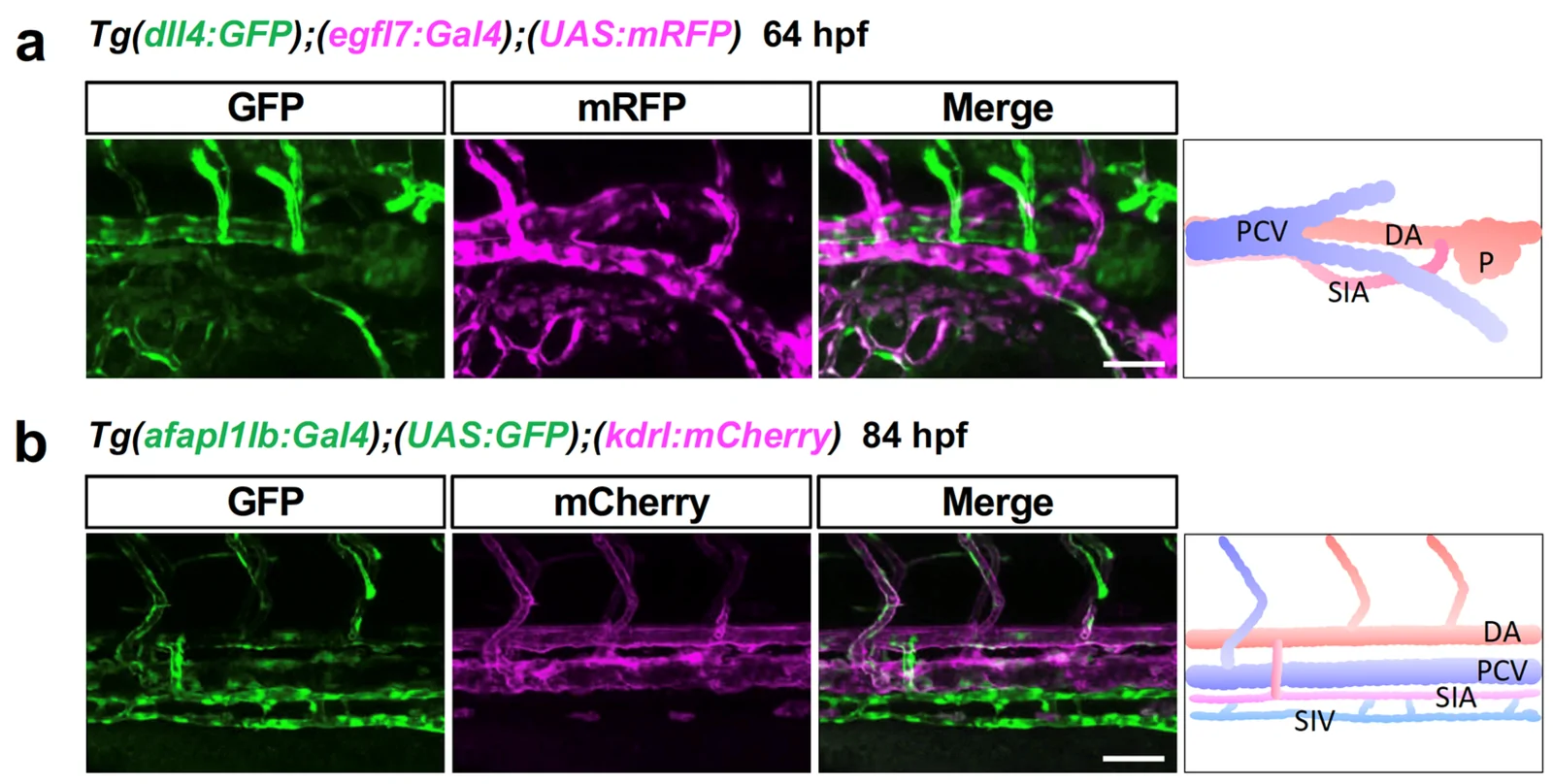
SIA-DA connections in the anterior and posterior regions. (**a**) Dorsolateral confocal images of the anterior SIA-DA connection of a 64 hpf *Tg(dll4:GFP);(egfl7:Gal4);(UAS:mRFP)* embryo. In the anterior region, *Tg(dll4:GFP)* expressed GFP in the DA and SIA, whereas *Tg(egfl7:Gal4);(UAS:mRFP)* expressed mRFP in the PCV. The *Tg(dll4:GFP)* line was useful for visualization of the anterior SIA-DA connection (see Supplemental Video S1). (**b**) Lateral confocal images of the posterior SIA-DA connection of an 84 hpf *Tg(afap1l1b:Gal4);(UAS:GFP)* embryo. In the posterior region, *Tg(afapl1b:Gal4);(UAS:GFP)* expressed GFP in the SIA, whereas *Tg(kdrl:mCherry)* expressed mCherry in the DA and PCV. The *Tg(afap1l1b:Gal4)* line was useful for visualization of the posterior SIA-DA connection (see Supplemental Video S2). Scale bar: 50 μm. P: Pronephric glomus.

To determine where the intestinal vasculature connects to the major axial vessels in systemic circulation, we conducted a comprehensive examination of SIA-DA junctions utilizing various endothelial reporter lines that differ in their expression patterns. We found the presence of SIA-DA connections in two distinct regions, the anterior and posterior. The anterior connection was observable using the *Tg(dll4:GFP)* line (Figure 2a and Supplemental Video S1), whereas the posterior connection was visualized using the *Tg(afap1l1b:Gal4);(UAS:GFP)* line (Figure 2b and Supplemental Video S2). These observations indicate that the SIA establishes connections with the DA in two spatially distinct regions.

### 3.3. Cellular Processes of the SIA-DA Connections in the Anterior and Posterior Regions

**Figure 3:**
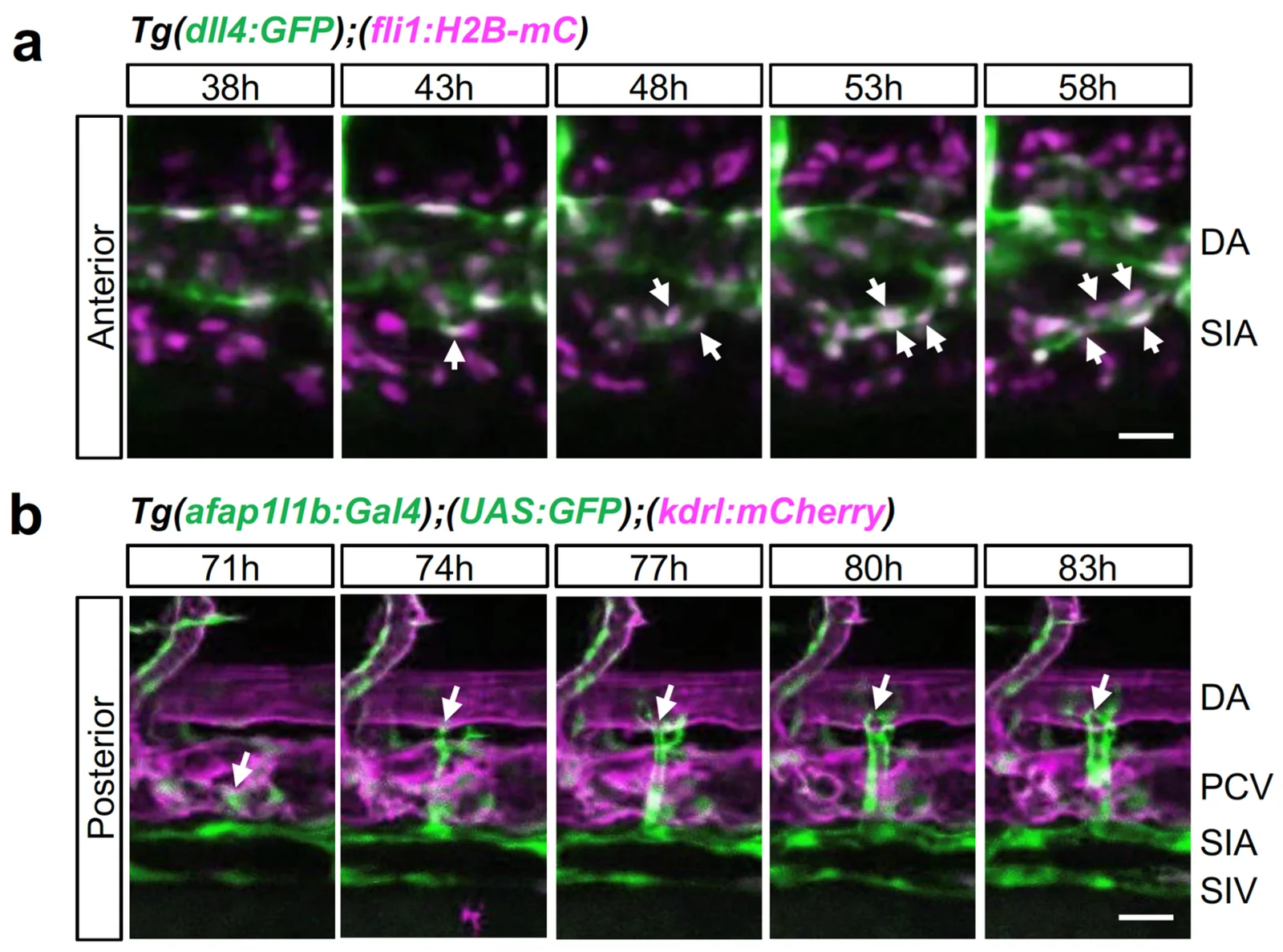
Cellular processes of the SIA-DA connections in the anterior and posterior regions. (**a**) Dorsolateral confocal images of the anterior SIA-DA connection in a *Tg(dll4:GFP);(fli1:H2B-mC)* embryo (see Supplemental Video S3). In the anterior region, *Tg(dll4:GFP)* marked the SIA-DA connection, whereas *Tg(fli1:H2B-mC)* labeled endothelial cell nuclei in the DA and PCV. Arrows indicate GFP- and mCherry-positive cells located outside the DA structure. The cells marked with arrows migrated out of the DA, proliferated, contacted the DA, and formed the SIA-DA connection between 38 and 58 hpf. (**b**) Lateral confocal images of posterior SIA-DA connection of a *Tg(afap1l1b:Gal4);(UAS:GFP);(kdrl:mCherry)* embryo (see Supplemental Video S4). In the posterior region, *Tg(afap1l1b:Gal4);(UAS:GFP)* showed minimal venous GFP expression and marked the SIA-DA connection and intestinal endothelial cells, whereas *Tg(kdrl:mCherry)* labeled the DA and PCV. The cells marked with arrows sprouted, contacted the DA and formed the lumenized SIA-DA connection between 71 and 83 hpf. Arrows indicate GFP-positive cells sprouted from intestinal endothelial cells. Scale bar: 10 μm.

In the anterior region, the SIA-DA connection was established by 64 hpf (Figure 2a). To examine how the anterior SIA-DA connection forms, we visualized this process using the *Tg(dll4:GFP);(fli1:H2B-mC)* line. Endothelial cells migrated out of the DA, proliferated the cells, made contact with DA and formed the SIA-DA connection between 38 and 58 hpf (Figure 3a and Supplemental Video S3). This observation suggested that the endothelial cells contributing to the anterior connection were derived from the DA rather than the PCV.

In the posterior region, the SIA-DA connection was established by 84 hpf (Figure 2b). To investigate the cellular process underlying the formation of the posterior SIA-DA connection, we visualized this process using the *Tg(afap1l1b:Gal4);(UAS:GFP);(kdrl:mCherry)* line. The posterior SIA-DA connection was initiated with sprouting from intestinal endothelial cells at around 71 hpf, followed by contact between endothelial sprouts and the DA, ultimately resulting in the formation of a lumenized connection at approximately 83 hpf (Figure 3b and Supplemental Video S4), indicating that the endothelial cells contributing to the posterior connection were derived from intestinal endothelial cells.

Together, these results reveal distinct cellular behaviors underlying the formation of anterior and posterior SIA-DA connections. In the anterior region, endothelial cells migrated from the DA to establish the SIA-DA connection, whereas in the posterior region, PCV-derived intestinal endothelial cells sprouted toward and subsequently connected to the DA. These findings indicate regionally distinct modes of intestinal–systemic vascular connection formation during zebrafish development.

### 3.4. Pharmacological Dissection of Signaling Pathways Required for the Posterior SIA-DA Connection

To begin to understand how these SIA-DA connections are regulated, we performed a pharmacological screen to examine the requirements of major signaling pathways involved in vascular development. Since the posterior SIA-DA connection can be more readily and consistently visualized than the anterior connection, we focused the inhibitor-based analyses on the posterior SIA-DA connection.

**Figure 4:**
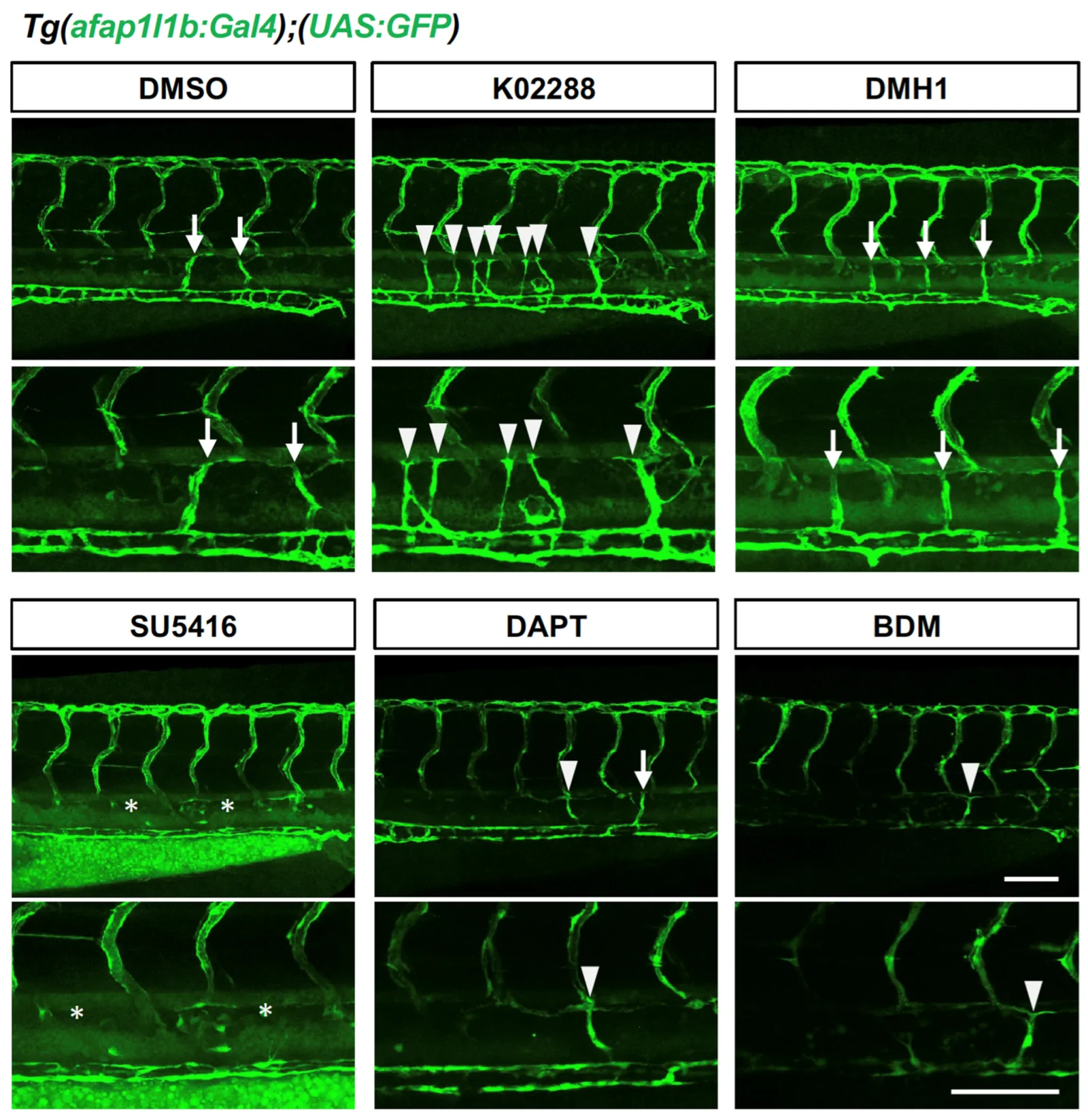
Pharmacological dissection of requirements for the posterior SIA-DA connection. Lateral confocal images of the posterior SIA-DA connection of 84 hpf *Tg(afap1l1b:Gal4);(UAS:GFP)* embryos treated with inhibitors. (**Lower**) panels show higher-magnification views from the (**upper**) panel images. Arrows indicate lumenized connections between the SIA and DA, whereas arrowheads indicate non-lumenized endothelial contacts with the DA. Asterisks indicate where connections or contacts with the DA are normally present. Scale bar: 50 μm.

We treated embryos from 60 to 84 hpf with inhibitors of VEGF (SU5416), ALK1/2 (K02288), ALK2 (DMH1) and Notch (DAPT) signaling pathways, as well as with BDM to abolish blood flow by inhibiting cardiac and skeletal muscle contraction, and observed the posterior SIA-DA connection at 84 hpf. Treatment with K02288, an ALK1/2 specific inhibitor, reduced the number of lumenized SIA–DA connections while increasing the number of non-lumenized endothelial contacts with the DA vessels compared to the control embryos, whereas DMH1, an ALK2-specific inhibitor, did not affect vessel lumenization (Figure 4). Inhibition of VEGF signaling using SU5416 completely abrogated the SIA-DA connection, while inhibition of Notch signaling with DAPT did not impair the SIA-DA connection (Figure 4). Additionally, the absence of blood flow induced by the BDM treatment led to a complete failure of the SIA-DA connection (Figure 4).

### 3.5. Genetic Requirement of Acvrl1 for the SIA-DA Connections

Among the signaling pathways implicated in the posterior SIA-DA connection through the pharmacological screen, we focused on ALK1 signaling in further genetic studies. We generated an *acvrl1* mutant fish line that lacks ACVRL1, a key receptor for ALK1 signaling [10,11,12,13]. The *acvrl1* homozygous mutants exhibited cranial vascular dilation and abnormal circulation, consistent with those reported in previous papers [12,13].

**Figure 5:**
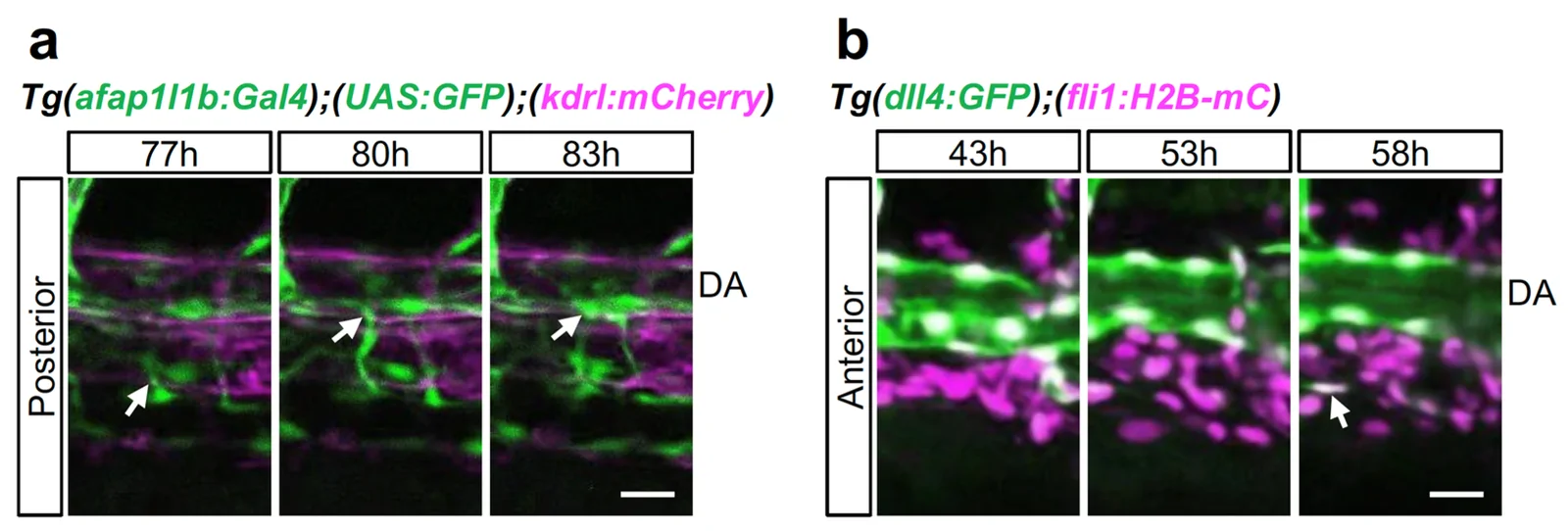
Genetic requirement of ALK1 signaling for the anterior and posterior SIA-DA connections. (**a**) Lateral confocal images of the posterior SIA-DA connection of the *acvrl1* mutant embryo with *Tg(afap1l1b:Gal4);(UAS:GFP);(kdrl:mCherry)* background (see Supplemental Video S5). Arrows indicate GFP-positive cells sprouted from intestinal endothelial cells. The corresponding wild-type cellular behavior under the same experimental conditions is shown in Figure 3b. (**b**) Dorsolateral confocal images of the anterior SIA-DA connection of the *acvrl1* mutant embryo with *Tg(dll4:GFP);(fli1:H2B-mC)* background (see Supplemental Video S6). An arrow indicates a GFP- and mCherry-positive cell located outside the DA structure. The corresponding wild-type cellular behavior under the same experimental conditions is shown in Figure 3a. Scale bar: 10 μm.

The SIA-DA connection in the posterior region of wild-type embryos consists of three consecutive steps, as visualized by time-lapse imaging (Supplemental Video S4 and Figure 3b). Endothelial cells sprouted from intestinal endothelial cells, contacted the DA, and formed the lumenized SIA-DA connection. In contrast, *acvrl1* mutants failed to form lumenized vessels (Supplemental Video S5 and Figure 5a). However, sprouting from intestinal endothelial cells and following SIA-DA contacts were largely unaffected, indicating that ALK1 signaling is specifically required for vessel lumenization at the later stages of posterior SIA–DA connection formation. In addition, we observed that *acvrl1* mutants failed to properly establish the anterior SIA-DA connection (Supplemental Video S6 and Figure 5b), although the precise morphological steps underlying this defect remain to be elucidated. Taken together, the pharmacological and genetic studies suggest that ALK1 signaling plays significant roles for SIA–DA connections during vascular development.

## 4. Discussion

**Figure 6:**
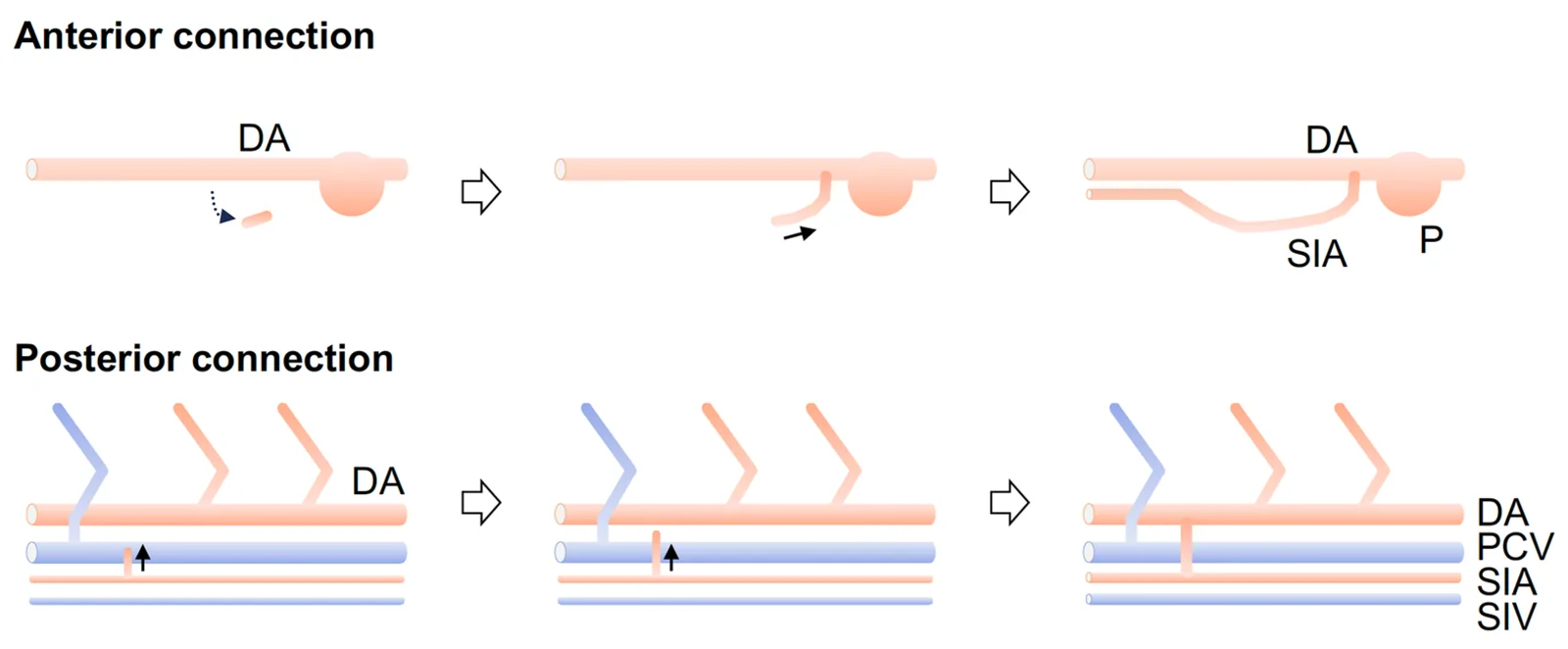
Schematic diagram of SIA-DA connection formations in the anterior and posterior regions. The SIA connects to the DA in two separate regions. The anterior SIA-DA connection forms first, followed by the posterior connection. Endothelial cells migrate out of the DA (dotted arrow) and form the anterior SIA-DA junction (arrow) (see Figure 3a and Supplemental Video S3), whereas intestinal endothelial cells sprout (arrow) and connect to the DA in the posterior region (see Figure 3b and Supplemental Video S4). P: Pronephric glomus.

In this study, we visualized how the developing intestinal vasculature connects to the pre-existing DA in zebrafish embryos and revealed two spatially and mechanistically distinct connection modes. Our findings indicate that the SIA connects to the DA in two separate regions: the anterior and posterior. Endothelial cells from the DA contributed to the anterior SIA-DA connection, whereas intestinal endothelial cells sprouted toward and connected to the DA in the posterior region (Figure 6). These findings uncover a previously unappreciated complexity in how intestinal circulation becomes integrated into the embryonic vascular system and suggest region-specific mechanisms underlying vascular anastomosis.

The presence of two distinct endothelial cell sources in the SIA-DA connections supports the idea that intestinal vascular integration proceeds in a stepwise manner. The anterior connection that utilizes DA-derived endothelial cells forms first, establishing the initial route of blood flow into the intestinal vasculature. The subsequent posterior connection, formed by angiogenic sprouts from PCV-derived intestinal endothelial cells, finalizes the circuit between the intestine and the systemic vasculature. This temporal sequence may serve to establish proper directional blood flow toward the developing liver and portal circulation. Our findings extend the previous notion that PCV-derived endothelial cells contribute to intestinal vessels such as the SIA and SIV through VEGF- and BMP-dependent sprouting [6,7,8]. Thus, differential cellular origins and temporal sequences likely coordinate the establishment of functional intestinal circulation during zebrafish development.

Our pharmacological and genetic analyses revealed that ALK1 signaling is required for proper formation of SIA-DA connections. In the posterior SIA-DA connection of *acvrl1* mutants, endothelial sprouts exhibited the capacity to reach and contact the DA, yet proper lumen formation was markedly impaired. These results suggest that ALK1 signaling is dispensable for the initial sprouting and targeting steps but essential for the subsequent lumenization and/or stabilization phase of anastomosis. Similar to its roles in the cranial and trunk vasculature [12,13,24], ALK1 signaling in the intestinal region is likely to promote endothelial maturation and the establishment of continuous lumens under hemodynamic forces. Consistently, inhibition of blood flow by the BDM treatment also disrupted posterior SIA–DA connection, suggesting that hemodynamic forces contribute to this process. Consistent with recent findings, blood flow regulates *acvrl1* expression during vascular remodeling [25]. In contrast, VEGF signaling is required for earlier phases of this process, as endothelial sprouts rarely reached the DA in the embryos treated with its inhibitor. These pharmacological analyses suggest that multiple signaling pathways and blood flow contribute to distinct steps of SIA–DA connection formation.

The posterior SIA-DA connection represents a mode of tip-to-tube anastomosis, where a sprouting endothelial tip connects to a pre-existing lumenized vessel. This configuration differs from the tip-to-tip fusion events that have been well characterized in the dorsal longitudinal anastomotic vessel and brain vasculature [26,27,28]. Although tip-to-tube anastomosis has not yet been mechanistically analyzed, our data suggest that ALK1 signaling may contribute to the stabilization and lumenal integration of the connecting tip. Recent live-imaging studies have highlighted the dynamic cellular processes underlying endothelial anastomosis and lumen formation [4]. VE-cadherin-mediated adhesion is known to be essential for endothelial junction stabilization during tip-to-tip anastomosis [29,30], and ALK1 may regulate similar adhesive or apical remodeling processes. Thus, the posterior SIA-DA junction provides a valuable in vivo model to investigate ALK1-dependent mechanisms of vascular connection.

The present study showed distinct endothelial behaviors in the anterior and posterior SIA-DA connections. Of note, the anterior connection also appeared to be disrupted in *acvrl1* mutants, while its precise characterization remains challenging in our pharmacological and genetic analyses. Although endothelial migration from the DA initiates anterior SIA-DA connection, the subsequent steps leading to its completion are not yet fully understood. Further investigation is warranted to elucidate commonalities and differences in the biological processes and regulatory mechanisms underlying the anterior and posterior SIA-DA connections.

## 5. Conclusions

In this study, we identified two regionally distinct modes of intestinal–systemic vascular connections in developing zebrafish and demonstrated that ALK1 signaling is indispensable for their proper establishment. Endothelial cells derived from different sources form the anterior and posterior SIA-DA connections sequentially to complete intestinal circulation. Our findings characterize key developmental aspects of intestinal vascular integration and may offer insights into ALK1-related vascular pathologies, such as arteriovenous malformations, in vertebrate systems.

## Data Availability

The data presented in this study are contained within the article or are available in Supplementary Data.

## Supplementary Materials

The following supporting information can be downloaded at: https://www.mdpi.com/article/10.3390/jdb14030037/s1, Figure S1: Genomic insertion sites of the *Tol2*-based gene trap construct in the *Tg(egfl7:Gal4)* and *Tg(afap1l1b:Gal4)* transgenic lines. Video S1: Time-lapse imaging of the anterior SIA-DA connection of a *Tg(dll4:GFP);(egfl7:Gal4);(UAS:mRFP)* embryo every 20 min at 64–72 hpf; Video S2: Time-lapse imaging of the posterior SIA-DA connection of a *Tg(afap1l1b):(Gal4;UAS:GFP);(kdrl:mCherry)* embryo every 30 min at 59–90 hpf; Video S3: Time-lapse imaging of the anterior SIA-DA connection of the control embryo with *Tg(dll4:GFP);(fli1:H2B-mC)* background every 10 min at 38–58 hpf; Video S4: Time-lapse imaging of the posterior SIA-DA connection of the control embryo with *Tg(afap1l1b:Gal4);(UAS:GFP);(kdrl:mCherry)* background every 30 min at 59–90 hpf; Video S5: Time-lapse imaging of the posterior SIA-DA connection of the *acvrl1* mutant embryo with *Tg(afap1l1b:Gal4);(UAS:GFP);(kdrl:mCherry)* background every 30 min at 59–90 hpf; Video S6: Time-lapse imaging of the anterior SIA-DA connection of the *acvrl1* mutant embryo with *Tg(dll4:GFP);(fli1:H2B-mC)* background every 10 min at 38–58 hpf.

## Abbreviations

The following abbreviations are used in this manuscript:

DA	Dorsal aorta
SIA	Supra-intestinal artery
SIV	Subintestinal vein
PCV	Posterior cardinal vein
ALK1	Activin receptor-like kinase 1
Acvrl1	activin A receptor-like type 1
VEGF	Vascular endothelial growth factor
BMP	Bone morphogenetic protein
ISV	Intersegmental vessel

## References

[1] Herbert S.P., Stainier D.Y. (2011). Molecular control of endothelial cell behaviour during blood vessel morphogenesis. Nat. Rev. Mol. Cell Biol..

[2] Trefts E., Gannon M., Wasserman D.H. (2017). The liver. Curr. Biol..

[3] Si-Tayeb K., Lemaigre F.P., Duncan S.A. (2010). Organogenesis and development of the liver. Dev. Cell.

[4] Okuda K.S., Hogan B.M. (2020). Endothelial Cell Dynamics in Vascular Development: Insights From Live-Imaging in Zebrafish. Front. Physiol..

[5] Phng L.K., Hogan B.M. (2024). Endothelial cell transitions in zebrafish vascular development. Dev. Growth Differ..

[6] Hen G., Nicenboim J., Mayseless O., Asaf L., Shin M., Busolin G., Hofi R., Almog G., Tiso N., Lawson N.D. (2015). Venous-derived angioblasts generate organ-specific vessels during zebrafish embryonic development. Development.

[7] Koenig A.L., Baltrunaite K., Bower N.I., Rossi A., Stainier D.Y., Hogan B.M., Sumanas S. (2016). Vegfa signaling promotes zebrafish intestinal vasculature development through endothelial cell migration from the posterior cardinal vein. Dev. Biol..

[8] Goi M., Childs S.J. (2016). Patterning mechanisms of the sub-intestinal venous plexus in zebrafish. Dev. Biol..

[9] Isogai S., Horiguchi M., Weinstein B.M. (2001). The vascular anatomy of the developing zebrafish: An atlas of embryonic and early larval development. Dev. Biol..

[10] Oh S.P., Seki T., Goss K.A., Imamura T., Yi Y., Donahoe P.K., Li L., Miyazono K., ten Dijke P., Kim S. (2000). Activin receptor-like kinase 1 modulates transforming growth factor-beta 1 signaling in the regulation of angiogenesis. Proc. Natl. Acad. Sci. USA.

[11] Urness L.D., Sorensen L.K., Li D.Y. (2000). Arteriovenous malformations in mice lacking activin receptor-like kinase-1. Nat. Genet..

[12] Roman B.L., Pham V.N., Lawson N.D., Kulik M., Childs S., Lekven A.C., Garrity D.M., Moon R.T., Fishman M.C., Lechleider R.J. (2002). Disruption of acvrl1 increases endothelial cell number in zebrafish cranial vessels. Development.

[13] Corti P., Young S., Chen C.Y., Patrick M.J., Rochon E.R., Pekkan K., Roman B.L. (2011). Interaction between alk1 and blood flow in the development of arteriovenous malformations. Development.

[14] Lawson N.D., Weinstein B.M. (2002). In vivo imaging of embryonic vascular development using transgenic zebrafish. Dev. Biol..

[15] Jin S.W., Beis D., Mitchell T., Chen J.N., Stainier D.Y. (2005). Cellular and molecular analyses of vascular tube and lumen formation in zebrafish. Development.

[16] Traver D., Paw B.H., Poss K.D., Penberthy W.T., Lin S., Zon L.I. (2003). Transplantation and in vivo imaging of multilineage engraftment in zebrafish bloodless mutants. Nat. Immunol..

[17] Yokota Y., Nakajima H., Wakayama Y., Muto A., Kawakami K., Fukuhara S., Mochizuki N. (2015). Endothelial Ca 2+ oscillations reflect VEGFR signaling-regulated angiogenic capacity in vivo. eLife.

[18] Mizoguchi T., Kawakami K., Itoh M. (2016). Zebrafish lines expressing UAS-driven red probes for monitoring cytoskeletal dynamics. Genesis.

[19] Asakawa K., Suster M.L., Mizusawa K., Nagayoshi S., Kotani T., Urasaki A., Kishimoto Y., Hibi M., Kawakami K. (2008). Genetic dissection of neural circuits by Tol2 transposon-mediated Gal4 gene and enhancer trapping in zebrafish. Proc. Natl. Acad. Sci. USA.

[20] Kawakami K., Abe G., Asada T., Asakawa K., Fukuda R., Ito A., Lal P., Mouri N., Muto A., Suster M.L. (2010). zTrap: Zebrafish gene trap and enhancer trap database. BMC Dev. Biol..

[21] Urasaki A., Morvan G., Kawakami K. (2006). Functional dissection of the Tol2 transposable element identified the minimal cis-sequence and a highly repetitive sequence in the subterminal region essential for transposition. Genetics.

[22] Jao L.E., Wente S.R., Chen W. (2013). Efficient multiplex biallelic zebrafish genome editing using a CRISPR nuclease system. Proc. Natl. Acad. Sci. USA.

[23] Urasaki A., Morishita S., Naka K., Uozumi M., Abe K., Huang L., Watase E., Nakagawa O., Kawakami K., Matsui T. (2019). Shootins mediate collective cell migration and organogenesis of the zebrafish posterior lateral line system. Sci. Rep..

[24] Rochon E.R., Menon P.G., Roman B.L. (2016). Alk1 controls arterial endothelial cell migration in lumenized vessels. Development.

[25] Anzell A.R., Kunz A.B., Donovan J.P., Tran T.G., Lu X., Young S., Roman B.L. (2024). Blood flow regulates acvrl1 transcription via ligand-dependent Alk1 activity. Angiogenesis.

[26] Herwig L., Blum Y., Krudewig A., Ellertsdottir E., Lenard A., Belting H.G., Affolter M. (2011). Distinct cellular mechanisms of blood vessel fusion in the zebrafish embryo. Curr. Biol..

[27] Lenard A., Ellertsdottir E., Herwig L., Krudewig A., Sauteur L., Belting H.G., Affolter M. (2013). In vivo analysis reveals a highly stereotypic morphogenetic pathway of vascular anastomosis. Dev. Cell.

[28] Betz C., Lenard A., Belting H.G., Affolter M. (2016). Cell behaviors and dynamics during angiogenesis. Development.

[29] Sauteur L., Affolter M., Belting H.G. (2017). Distinct and redundant functions of Esama and VE-cadherin during vascular morphogenesis. Development.

[30] Hübner K., Cabochette P., Diéguez-Hurtado R., Wiesner C., Wakayama Y., Grassme K.S., Hubert M., Guenther S., Belting H.G., Affolter M. (2018). Wnt/β-catenin signaling regulates VE-cadherin-mediated anastomosis of brain capillaries by counteracting S1pr1 signaling. Nat. Commun..

