# Supplementary material for: Regionally Distinct Modes of Intestinal–Systemic Vascular Connections in Developing Zebrafish
Source: J Dev Biol. 2026 Aug 14;14(3):37. doi: 10.3390/jdb14030037 (PMC13511000; doi:10.3390/jdb14030037)
Supplement: Supplementary file 1 [file jdb-14-00037-s001.zip › Figure S1.pdf]

Supplementary Figure

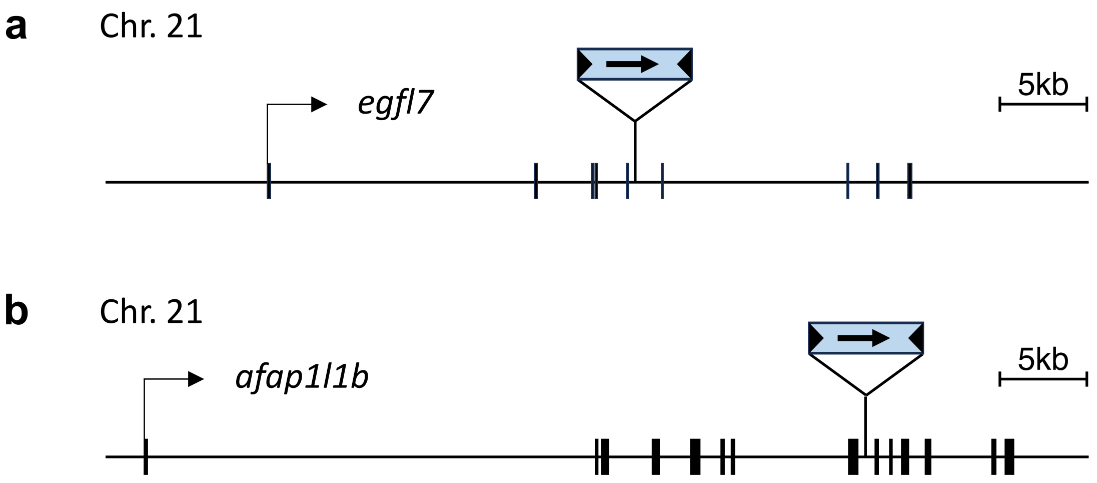

Figure S1. Genomic insertion sites of the *Tol2*-based gene trap construct in the *Tg(egfl7:Gal4)* and *Tg(afap1l1b:Gal4)* transgenic lines. (a) Schematic diagram of the genomic insertion site of the *Tol2*-based gene trap construct in the *Tg(egfl7:Gal4)* line. The construct is integrated into an intron of *egfl7* on chromosome 21. (b) Schematic diagram of the genomic insertion site of the *Tol2*-based gene trap construct in the *Tg(afap1l1b:Gal4)* line. The construct is integrated into an intron of *afap1l1b* on chromosome 21. Black boxes and blue boxes represent exons and the *Tol2*-based gene trap constructs, respectively.
